# Pathogens distribution and drug resistance in patients with acute cerebral infarction complicated with diabetes and nosocomial pulmonary infection

**DOI:** 10.1186/s12879-019-4142-9

**Published:** 2019-07-10

**Authors:** Yu-Xin Liu, Qiu-Mei Cao, Bing-Chen Ma

**Affiliations:** 1 Department of Emergency at Beijing Tongren Hospital of Capital Medical University, Beijing, 100176 China; 2 Department of General Medicine at Beijing Tongren Hospital of Capital Medical University, No. 2 of Western South Road of Yizhuang, Daxing District, Beijing, 100176 China

**Keywords:** Acute cerebral infarction, Diabetes mellitus(DM), Pulmonary infection, Pathogen, Drug-resistance

## Abstract

**Background:**

This study aims to investigate the pathogen distribution and drug resistance in patients with acute cerebral infarction complicated with diabetes mellitus and nosocomial pulmonary infection.

**Methods:**

From August 2015 to December 2017, 172 pathogenic bacterial strains from patients with acute cerebral infarction complicated with diabetes mellitus and nosocomial pulmonary infection in our hospital were identified, and the drug sensitivity was analyzed.

**Results:**

Among these 172 strains of pathogenic bacteria, gram negative bacteria was the main cause of pulmonary infection in hospitalized patients with acute cerebral infarction, accounting for 75.6% of all pathogens. Furthermore, 80% of diabetic patients with cerebral infarction had lung infection induced by gram negative bacteria, which was significantly higher than that in non-diabetic patients (72.2%). Moreover, the drug resistance rate in the diabetic group (68.3%) was significantly higher than that in the non-diabetic group (54.3%). Gram positive bacteria accounted for 19.1% of all pathogenic bacteria. The infection rate of gram-positive bacteria in diabetic patients with cerebral infarction was 14.7%, which was lower than that in the non-diabetic group (22.6%). The drug-resistance rate was higher in the diabetic group (45.5%) than in the non-diabetic group (28.2%). Furthermore, the fungal infection rate in patients with lung infection in these two groups was 5.3 and 5.2%, respectively, and fungi presented with high sensitivity to commonly used antifungal agents.

**Conclusion:**

In patients with acute cerebral infarction complicated with diabetes mellitus and nosocomial pulmonary infection, the majority of pathogens are multidrug-resistant gram negative bacilli. Pathogen culture should be conducted as soon as possible before using antibiotics, and antimicrobial agents should be reasonably used according to drug sensitivity test results.

## Background

With the change in people’s diet and lifestyle, and the aged tendency of population, the prevalence, morbidity and mortality of cerebral infarction in China have increased annually. Therefore, cerebral infarction endangers the health and burdens the family of countless people. Once cerebral infarction is associated with pulmonary infection, this would first affect the treatment of the primary disease, and subsequently increase the mortality and disability rate, and increase hospitalization days. Furthermore, this would also enhance the economic burden of the patient’s family and society. Cerebral infarction mainly occurs in the elderly, or in patients with hypertension or diabetes, *and a high glucose environment in diabetic patients is beneficial for the proliferation of bacteria.* Therefore, diabetic patients are more prone to pulmonary infection [1, 2]. In the present study, the pathogenic bacteria and drug resistance of pulmonary infection in hospitalized patients with acute cerebral infarction (ACI), especially patients with diabetes mellitus, were analyzed and compared, in order to help reduce the mortality and disability rate of lung infection in the future.

## Methods

### General information

From August 2015 to December 2017, 1093 ACI patients were admitted to the neurology ward of Beijing Tongren Hospital, among which 152 patients were detected with pulmonary infection 2–7 days after the diagnosis of ACI. Thus, the rate of pulmonary infection among all patients with ischemic stroke was 13.9%.

In 152 patients with pneumonia, 82 patients were male and 70 patients were female, and the age of these patients ranged within 53–95 years old. A total of 172 strains of effective pathogenic bacteria were cultured. The clinical data of these patients were retrospectively analyzed. Then, these patients were divided into two groups, according to the presence of diabetes: diabetic group and non-diabetic group. The diagnostic criteria for diabetes were based on the 2017 Guideline for Diabetes Diagnosis and Treatment in the United States [3]. All patients with cerebral infarction were confirmed by head magnetic resonance imaging (MRI) or computed tomography (CT), and met the diagnostic criteria of the 2018 Early Management Guidelines for Acute Ischemic Stroke Patients [4]. The following infarction patterns were identified in these 152 patients: anterior circulation infarction (*n* = 86), posterior circulation infarction (*n* = 37), and anterior/posterior mixed infarction (*n* = 29).

Diagnostic criteria for pulmonary infection: absence of pulmonary infection before stroke, the onset occurred 48 h after admission, and the following conditions were observed: (1) fever, body temperature of > 38 °C; (2) purulent airway secretions; (3) peripheral blood leucocyte count was > 10 × 10^9^/L or < 4 × 10^9^ [5]. The presence of two or more of the above three clinical symptoms, with newly or progressive infiltration, consolidation, or ground-glass shadows on chest X-ray or CT films, confirms the diagnosis of pulmonary infection. Sixty patients (39.5%) suffered from dysphagia in this study, among which 16 had disorders of consciousness (10.5%) and 44 experienced cough (29.0%). Exclusion criteria: (1) patients with pulmonary infection before onset of stroke or patients with chronic pulmonary disease; (2) patients with mental disorders or language disorders; (3) patients combined with other organ dysfunction or malignant tumors.

### Specimen collection and pathogen culture

Sputum specimens were collected in the morning. Patients gargled once with normal saline before specimen collection, and forcefully coughed up the deep sputum. The sputum was spat into a sterile culture box for the test, or the sputum specimen was obtained from the tracheal sheath. All included specimens were eligible lower respiratory tract secretions (neutrophils > 25/low power microscopic visual field, epithelial cell count < 10/low power microscopic visual field, or the ratio of these two was > 2.5:1). Isolation of pathogenic bacteria: The isolation of strains was carried out according to the National Regulations for Clinical Laboratory Practice. The bacteria were identified using a MALDI-TOFMS mass spectrometer. The drug sensitivity test was carried out using the French VITEK-2 compact microbial identification system and the Kirby-Bauer disk diffusion method according to the CLSI guidelines [Add reference: Clinical and Laboratory Standards Institute. Performance standards for antimicrobial susceptibility testing. CLSI M100-S22].

### Statistical methods

Statistical software SPSS 19 was used to analyze the data. Measurement data were expressed as mean ± standard deviation (x ± SD), and compared between groups conducted using *t*-test. Count data were expressed in percentage (%), and compared using Chi-square test. *P* < 0.05 was considered statistically significant.

## Results

### The distribution of pathogens in all nosocomial infections in the hospital

The data of pathogen distribution of all nosocomial infections in the hospital from 2015 to 2017 were summarized in Table 1.

**Table 1:** The distribution of pathogens in all nosocomial infections in the hospital

Key pathogenic bacteria	Year		
	2015 (%)	2016 (%)	2017 (%)
*Escherichia coli*	10.14	18.01	17.50
*Pseudomonas aeruginosa*	15.65	16.91	13.88
*Klebsiella pneumoniae*	9.57	10.29	12.15
Baumanii	8.99	7.35	7.38
*Candida albicans*	4.93	No	5.42
*Staphylococcus aureus*	3.48	6.99	5.21
*Staphylococcus epidermidis*	26.38	No	77.0
*Enterococcus faecium*	4.64	5.51	4.34

### Comparison of general data

Comparison of cerebral infarction between the diabetic group and non-diabetic group: the difference in age between these two groups were not statistically significant, the difference in the proportion of gender was not statistically significant, and the difference in the degree of (NIHSS score) between these two groups was not statistically significant (Table 2).

**Table 2:** The characteristics of cerebral infarction patients in the diabetic and non-diabetic groups

Groups	Diabetic group (*n* = 60)	Non-diabetic group (*n* = 92)	P
Age	75.35 ± 6.224	74.91 ± 10.799	0.752
Gender(Male/Female)	32/28	50/42	0.959
NIHSS score	17.03 ± 1.149	16.8 ± 1.260	0.259

### Sputum culture results

A total of 208 qualified sputum specimens were sent for testing, 172 strains of pathogenic bacteria were detected, and the detection rate of pathogens was 82.7%. Patients in the diabetic group had 60 strains of Gram-negative bacteria (80%), 11 strains of Gram-positive bacteria (14.7%), and four strains of fungi (5.3%). Patients in the non-diabetic group had 70 strains of Gram-negative bacteria (72.2%), 22 strains of Gram-positive bacteria (22.6%), and five strains of fungi (5.2%). The distribution and proportion of pathogenic bacteria are presented in Table 3.

**Table 3:** The distribution and proportion of pathogenic bacteria

Pathogenic bacteria	Diabetic group	Non-diabetic group
	Number of case(Constituent ratio %)	Number of case(Constituent ratio %)
Gram-negative bacterium	60 (80.0)	70 (72.2)
Baumanii	27 (36.0)	32 (33)
Klebsiella Pneumoniae	7 (9.3)	5 (5.2)
Pseudomonas Aeruginosa	13 (17.3)	19 (19.5)
Escherichia coli	7 (9.3)	6 (6.2)
*Enterobacter cloacae*	2 (2.7)	1 (1)
Acinetobacter Pistorii	1 (1.3)	4 (4.1)
Others	3 (4.0)	3 (3.1)
Gram-positive Bacterium	11 (14.7)	22 (22.6)
Staphylococcus aureus	5 (6.7)	15 (15.5)
*Streptococcus pneumoniae*	4 (5.3)	4 (4.1)
Enterococcus Faecium	2 (2.7)	3 (3.1)
Fungus	4 (5.3)	5 (5.2)
Candida albicans	2 (2.6)	3 (3.1)
Candida lucidum	1 (1.3)	2 (2.1)
Total	75	97
Statistic	x^2^ = 21.253	P = 0.001

The proportion of bacteria in these two groups was statistically analyzed (*×* ^2^ = 21.253, *P* = 0.001). In the diabetic group, the proportion of Gram-negative bacteria was higher, when compared with the non-diabetic group, while the proportion of Gram-positive bacteria was lower, when compared with the non-diabetic group, and the difference in the proportion of cultured fungi was not statistically significant. This suggests that the proportion of Gram-positive bacteria in the pathogen was higher in diabetic patients with cerebral infarction.

The drug resistance rate of all strains was statistically analyzed. In the diabetic group, the drug-resistance rate of Gram-negative bacteria was 68.3%, and the drug-resistance rate of Gram-positive bacteria was 45.5%. In the non-diabetic group, the drug-resistance rate of Gram-negative bacteria was 54.3%, and the drug-resistance rate of Gram-positive bacteria was 28.2%. No drug-resistant fungi were found during culture in these two groups. The drug-resistance rates are presented in Tables 4 and 5. The drug-resistance rates of Gram-negative and Gram-positive bacteria were higher in the diabetic group than in the non-diabetic group (Table 6; *×* ^2^ = 8.817, *P* = 0.003).

**Table 4:** Resistance rate of major gram-negative bacilli to commonly used drugs(%)

Name of drug	Diabetic group	Non-diabetic group
Imipenem	38.4	25.9
Piperacillin and Sulbactam	68.2	55.9
Piperacillin and Tazobactam	49.7	59.6
ceftazidime	82.5	54.6
ceftriaxone	91.7	78.9
Ciprofloxacin	95.6	66.1
amikacin	50.8	42.7
Trimethoprim/sulfonamide	85.6	64.1
gentamicin	83.3	70.3
levofloxacin	61.6	50.8
tobramycin	44	28.6
drug-resistance rates	68.3	54.3

**Table 5:** Resistance rate of major gram-positive cocci to antibiotics(%)

Name of drug	Diabetic group	Non-diabetic group
levofloxacin	0	0
levofloxacin	70.5	38.2
linezolid	0	0
vancomycin	0	0
penicillin	100	80
tetracycline	51.6	36.3
Piperacillin and Sulbactam	60.9	50.6
erythrocin	58.3	15.4
oxacillin	83.8	62.6
rifampicin	35.4	12.9
cefazolin	82.7	60.3
moxifloxacin	25.2	5.6
Trimethoprim/sulfonamide	23.3	4.8
drug-resistance rates	45.5	28.2

**Table 6:** Comparison of drug resistance rates between the two groups

	Diabetic group	Non-diabetic group
Drug resistance rate of gram-negative bacilli	68.3%	54.3%
Drug resistance rate of gram-positive cocci	45.5%	28.2%
Statistic	x^2^ = 8.817	P = 0.003

## Discussion

Pulmonary infection is a serious complication of stroke, which significantly increases mortality rate and medical costs, and affects the prognosis of stroke patients [6, 7]. Pulmonary infection in patients with cerebral infarction is induced through multiple factors: (1) Most patients with cerebral infarction are elderly individuals, who have many underlying diseases, and often have different degrees of hypofunction of the lung and chronic respiratory diseases. (2) Stroke can directly and indirectly affect hypothalamic function, cause autonomic nerve dysfunction in internal organs, and induce pulmonary arterial hypertension and damage to pulmonary capillaries, resulting in pulmonary blood stasis and pulmonary edema [8, 9], and this is the pathological basis of pulmonary infection. (3) Patients with ACI often have true bulbar paralysis or pseudobulbar paralysis, namely, dysphagia, where a long-term indwelling nasal feeding tube is needed. The nasal feeding tube increases the risk of aspiration [10, 11]. This causes consciousness disorder, coughing and vomiting reflexes to be further impaired, and prevents airway secretions or inhaled objects from being easily excreted, leading to pulmonary infections. (4) Stroke leads to nervous-endocrine-immune regulation disorders, which inhibit the T-lymphocyte-assisted differentiation of other immune cells and regulate immune response [12, 13], inducing pulmonary infection. (5) Long-term lying in the bed and gastrointestinal dysfunction can result to poor indigestion, leading to malnutrition. Furthermore, decreased immunity is also a risk factor for pulmonary infection.

Pulmonary infection is also one of the serious complications of diabetes, and is also the main cause of death in diabetics. First, increased glycosylated hemoglobin in diabetic patients can cause the oxygen dissociation curve to shift to the left, which is not conducive for oxygen release. Moreover, in diabetic patients, the basement membrane of pulmonary capillaries thickens [14], pulmonary surfactants decrease and the ventilation/blood flow ratio becomes imbalanced, while decreased blood oxygen leads to lung infection. Second, hyperglycemia in diabetes often leads to elevated blood osmotic pressure, and decreased chemotaxis, phagocytosis and self-defense ability of granulocytes, which decreases the anti-infection ability [15]. In addition, diabetic patients often have immune dysfunction as a result of respiratory immune function deficiency, and accordingly develop repeated respiratory infection [16, 17].

Both type-2 diabetes mellitus and cerebral infarction can easily cause infection. When pulmonary infection occurs in diabetic patients at the acute stage of cerebral infarction, it will initially affect the treatment of the primary disease and increase the mortality and disability rates, and subsequently prolong the length of hospital stay, aggravating the economic burden of the family and society.

Pathogen distribution and drug resistance in pulmonary infection in hospitalized patients with ACI complicated with and without diabetes mellitus were analyzed. The results revealed that Gram-negative bacteria were the main cause of pulmonary infection in hospitalized patients with ACI, accounting for 75.6% of all pathogens. This is similar to the result reported by Zongding Zeng [18]. Furthermore, 80% of diabetic patients with cerebral infarction had lung infection induced by Gram-negative bacteria, which was significantly higher than that in non-diabetic patients (72.2%). Moreover, the drug resistance rate in the diabetic group (68.3%) was significantly higher than that in the non-diabetic group (54.3%). Gram-negative bacteria in these two groups were mainly Bauman’s Acinetobacter and *Pseudomonas aeruginosa*, which are highly resistant to third-generation cephalosporin, ciprofloxacin, trimethoprim/sulfanilamide and gentamicin, and sensitive to imipenem, tobramycin and amikacin. Gram-positive bacteria accounted for 19.1% of all pathogenic bacteria. Furthermore, the infection rate of Gram-positive bacteria in diabetic patients with cerebral infarction was 14.7%, which was lower than that in the non-diabetic group (22.6%). The Gram-positive bacteria were mainly *Staphylococcus aureus*, followed by *Streptococcus pneumoniae*. The drug resistance rate was higher in the diabetic group (45.5%) than in the non-diabetic group (28.2%), and the bacteria were mainly resistant to penicillins, cefamycin and levofloxacin, but were not resistant to vancomycin and linezolid. The fungal infection rate in patients with lung infection in these two groups was 5.3 and 5.2%, respectively, and fungi presented with high sensitivity to commonly used antifungal agents.

When pulmonary infection occurs in ACI patients, the early rational selection of sensitive antibiotics directly affects the prognosis of the disease. Before the pathogen is identified, sensitive antibiotics can be empirically selected according to the infectious flora and drug resistance characteristics of diabetic patients and non-diabetic patients. Before antibiotic treatment, attention should be given when taking and sending qualified specimens. After obtaining reliable results of the bacterial culture and drug sensitivity test, antibiotics with high sensitivity were selected according to the characteristics of the disease and type of pathogenic bacteria [19], in order to improve the pertinence and effectiveness of the anti-infective therapy, and reduce the formation of drug-resistant strains.

## Conclusions

For patients with ACI, in addition to actively treating the primary disease, early prevention, early diagnosis and active treatment should be directly given against high-risk factors, such as diabetes, hypertension and heart disease, and respiratory tract management and drug resistance monitoring should be strengthened, in order to suppress the growth trend of drug resistance of pathogens, minimize the incidence of pulmonary infection, improve the effectiveness of the pulmonary infection treatment, and improve the prognosis of patients.

## Data Availability

The datasets used and/or analysed during the current study available from the corresponding author on reasonable request.

## Abbreviations

ACI: Acute cerebral infarction
CT: Computed tomography
DM: Diabetes mellitus
MRI: Magnetic resonance imaging
